# Essential Genes for *In Vitro* Growth of the Endophyte Herbaspirillum seropedicae SmR1 as Revealed by Transposon Insertion Site Sequencing

**DOI:** 10.1128/AEM.02281-16

**Published:** 2016-10-27

**Authors:** Federico Rosconi, Stefan P. W. de Vries, Abiyad Baig, Elena Fabiano, Andrew J. Grant

**Affiliations:** a Departamento de Bioquímica y Genómica Microbianas, IIBCE, Montevideo, Uruguay; b Department of Veterinary Medicine, University of Cambridge, Cambridge, United Kingdom; University of Tartu

## Abstract

The interior of plants contains microorganisms (referred to as endophytes) that are distinct from those present at the root surface or in the surrounding soil. Herbaspirillum seropedicae strain SmR1, belonging to the betaproteobacteria, is an endophyte that colonizes crops, including rice, maize, sugarcane, and sorghum. Different approaches have revealed genes and pathways regulated during the interactions of H. seropedicae with its plant hosts. However, functional genomic analysis of transposon (Tn) mutants has been hampered by the lack of genetic tools. Here we successfully employed a combination of *in vivo* high-density mariner Tn mutagenesis and targeted Tn insertion site sequencing (Tn-seq) in H. seropedicae SmR1. The analysis of multiple gene-saturating Tn libraries revealed that 395 genes are essential for the growth of H. seropedicae SmR1 in tryptone-yeast extract medium. A comparative analysis with the Database of Essential Genes (DEG) showed that 25 genes are uniquely essential in H. seropedicae SmR1. The Tn mutagenesis protocol developed and the gene-saturating Tn libraries generated will facilitate elucidation of the genetic mechanisms of the H. seropedicae endophytic lifestyle.

**IMPORTANCE** A focal point in the study of endophytes is the development of effective biofertilizers that could help to reduce the input of agrochemicals in croplands. Besides the ability to promote plant growth, a good biofertilizer should be successful in colonizing its host and competing against the native microbiota. By using a systematic Tn-based gene-inactivation strategy and massively parallel sequencing of Tn insertion sites (Tn-seq), it is possible to study the fitness of thousands of Tn mutants in a single experiment. We have applied the combination of these techniques to the plant-growth-promoting endophyte Herbaspirillum seropedicae SmR1. The Tn mutant libraries generated will enable studies into the genetic mechanisms of H. seropedicae-plant interactions. The approach that we have taken is applicable to other plant-interacting bacteria.

## INTRODUCTION

Plants rely on beneficial interactions with their microbiota for nutrient availability, growth promotion, and suppression of disease. The plant interior, referred to as the endosphere, has been shown to contain a distinct microbiome that is less diverse than those from the rhizoplane (the root surface) and the rhizosphere (a narrow zone of soil subject to the influence of living roots) (1). Microorganisms that colonize the endosphere are referred to as endophytes (2, 3); these include all microorganisms that for all or part of their lifetimes colonize internal plant tissues (4).

The knowledge of plant-bacterial endophyte interactions at the genetic and molecular levels has increased due to the use of suitable (laboratory-controlled) biological models. A model endophyte is Herbaspirillum seropedicae, a member of the Betaproteobacteria subclass, which includes many plant-associated bacteria such as species of the genera Azoarcus, Burkholderia, and Ralstonia (5). Several characteristics make H. seropedicae a suitable model endophyte (6), i.e., (i) it provides fixed nitrogen for important agroeconomic cultivars, (ii) it is genetically tractable, (iii) it has mechanisms of plant growth promotion other than nitrogen fixation, (iv) it has a wide range of plant hosts, (v) culturable bacteria are not isolated from soil and are isolated only from inside plants (7, 8), and (vi) there are publicly available genome sequences (8). Some isolates of H. seropedicae have been described as being pathogenic in plants, although this may be the result of the host being unable to control colonization, and there have also been reports that it can be an opportunistic pathogen in immunocompromised individuals (9, 10). The most well-studied H. seropedicae strains, SmR1 and Z67, have been tested in different plant species without symptoms of disease (11).

Recently, transcriptomic and proteomic approaches have identified genes and pathways that are regulated during the interactions of H. seropedicae with different plant hosts (12–14). In addition, comparative genomics and metagenomics studies have shown that certain functions, e.g., nutrient transport systems, type IV conjugal DNA-protein transfer secretion systems, plant growth promotion genes, and iron uptake systems, are overrepresented in the genomes of bacterial endophytes, compared to rhizospheric or soil bacteria (4, 15–17). Gene inactivation/deletion studies have shown that lipopolysaccharide (LPS) production is essential for effective H. seropedicae attachment to maize roots (18), and high-affinity iron uptake mechanisms contribute to the competitive fitness of H. seropedicae inside host plants (19).

Compared to gene expression and comparative genomics studies, high-throughput functional analyses of endophyte-plant interactions have lagged. In recent years, there has been much progress in the application of transposon (Tn)-based gene inactivation methods in combination with massively parallel sequencing of Tn insertion sites, e.g., Tn insertion site sequencing (Tn-seq) and related techniques (20–22), which have advanced, and continue to advance, the characterization of bacterium-host interactions.

In this study, we successfully employed *in vivo* mariner Tn mutagenesis in H. seropedicae strain SmR1 and characterized the resulting Tn mutants by Tn-seq. The resulting data set was used to identify the genes that, upon inactivation, have detrimental effects on fitness during *in vitro* growth and survival, i.e., essential genes.

## MATERIALS AND METHODS

### Bacterial strains, media, and growth conditions.

H. seropedicae SmR1 was routinely cultured at 30°C in TY medium (tryptone, 5 g/liter; yeast extract, 3 g/liter; CaCl_2_, 0.1 g/liter) (5). Escherichia coli strains NEB 5-alpha (New England BioLabs), TransforMax EC100D *pir*^+^ (Epicentre), and SM10-λpir were cultured at 37°C in Luria-Bertani (LB) broth (23). Where necessary for selection of plasmids, the medium was supplemented with ampicillin (50 μg/ml), kanamycin (50 μg/ml), or tetracycline (10 μg/ml) as appropriate (24, 25). For selection of H. seropedicae SmR1 Tn mutants, TY medium was supplemented with streptomycin (100 μg/ml) and either kanamycin (200 μg/ml) or tetracycline (10 μg/ml) as appropriate. The bacterial strains and plasmids used in this study are listed in Table 1.

**TABLE 1:** Bacterial strains and plasmids used in this study

Strain and plasmid	Relevant genotype and/or description^*a*^	Source or reference
Herbaspirillum seropedicae SmR1	Wild type; spontaneous streptomycin-resistant mutant of strain Z78	5
Escherichia coli		
NEB 5-alpha	Subcloning efficiency DH5α-derived competent cells; *fhuA2* Δ(*argF-lacZ*)*U169 phoA glnV44* Φ80Δ(*lacZ*)*M15 gyrA96 recA1 relA1 endA1 thi-1 hsdR17*	New England Biolabs
TransforMax EC100D *pir*^+^	Electrocompetent cells constitutively expressing *pir* gene; *F*^−^ *mcrA* Δ(*mrr-hsdRMS-mcrBC*) ϕ80d*lacZ*Δ*M15* Δ*lacX74 recA1 endA1 araD139* Δ(*ara*, *leu*)*7697 galU galK* λ− *rpsL* (Str^R^) *nupG pir^+^*(*DHFR*)	Epicentre
SM10-λpir	Donor strain carrying transfer genes of broad host range; IncP-type plasmid RP4, Km^r^; *thi-1 thr leu tonA lacY supE recA*::*RP4-2-Tc*::*Mu pir*	23
Plasmids		
pBR322	Cloning vector; Amp^r^, Tc^r^	24
pMiniT	Cloning vector; Amp^r^	New England Biolabs
pFRC002	*tetA* gene from pBR322 cloned in pMiniT; Amp^r^, Tc^r^	This work
pSAM_R1	Suicide mobilizable vector; Amp^r^, Km^r^, *himar1-C9*	25
pSAM_R5	*tetA* gene from pFRC002 cloned in pSAM_R1; Amp^r^, Tc^r^	This work

a Amp, ampicillin; Km, kanamycin; Tc, tetracycline.

### Recombinant DNA techniques.

Standard methods were used for molecular cloning (26). Chromosomal and plasmid DNA purification, DNA modification, and ligations were performed using commercial kits (from Qiagen, Thermo Scientific, or New England BioLabs), according to the manufacturers' instructions. DNA concentrations were measured using a Nanodrop ND-1000 spectrophotometer (Thermo Scientific). PCR primers were purchased from Sigma-Genosys. Thermal cycling was performed in a GeneAmp PCR System 9700 (PE Applied Biosystems) or T100 thermal cycler (Bio-Rad). Thermal cycling conditions were 96°C for 2 min, 30 cycles of 96°C for 1 min, 55 to 60°C for 1 min, and 72°C for 30 s/kb, and finally extension at 72°C for 5 min.

### Generation of Tn mutant libraries.

For construction of Tn mutants, we used either (i) plasmid pSAM_R1 (25), which contained the mariner Tn with the kanamycin resistance gene *nptII* and the *Himar1_C9* transposase gene under the control of the *rpoD* promoter of the alphaproteobacterium Rhizobium leguminosarum, or (ii) plasmid pSAM_R5, in which we replaced the *nptII* gene with the *tet* gene of plasmid pBR322, flanked by mariner-specific inverted repeats. The *tetA* gene from pBR322 was amplified with primers Tet_FW1_XhoI and Tet_RV1_XbaI (details of the oligonucleotides used in this study are presented in Table 2). PCR amplicons were cloned into pMiniT using the NEB PCR cloning kit (New England BioLabs), generating plasmid pFRC002. This plasmid was digested with XhoI and XbaI (New England BioLabs), and the fragment released was gel purified and cloned into the same restriction enzyme sites of pSAM_R1, generating pSAM_R5. The sequences of these plasmids were confirmed by Sanger sequencing (Source BioScience). Subsequently, the plasmids were transformed into E. coli TransforMax EC100D *pir*^+^ (Epicentre).

**TABLE 2:** Primer sequences used in this study^*a*^

Name	Characteristics	Sequence (5′ to 3′)
Primers		
Tet_FW1_XhoI	For amplification of *tetA* gene	CTCGAGTCTCATGTTTGACAGCTTATCATCG
Tet_RV1_XbaI	For amplification of *tetA* gene	TCTAGAGTTTGCGCATTCACAGTTCTCCG
Km_RV2	For identification of Tn insertion sites by single-primer PCR	CGTGCAATCCATCTTGTTCAATC
Oligonucleotides for Tn-seq		
PBGSF29 ATCACG	Adapter primer with ATCACG barcode	TTCCCTACACGACGCTCTTCCGATCTATCACGNN
PBGSF30 ATCACG	Adapter primer with ATCACG barcode	P-CGTGATAGATCGGAAGAGCGTCGTGTAGGGAAAGAGT-P
PBGSF29 CGATGT	Adapter primer with CGATGT barcode	TTCCCTACACGACGCTCTTCCGATCTCGATGTNN
PBGSF30 CGATGT	Adapter primer with CGATGT barcode	P-ACATCGAGATCGGAAGAGCGTCGTGTAGGGAAAGAGT-P
PBGSF29 TGACCA	Adapter primer with TGACCA barcode	TTCCCTACACGACGCTCTTCCGATCTTGACCANN
PBGSF30 TGACCA	Adapter primer with TGACCA barcode	P-TGGTCAAGATCGGAAGAGCGTCGTGTAGGGAAAGAGT-P
PBGSF29 CTTGTA	Adapter primer with CTTGTA barcode	TTCCCTACACGACGCTCTTCCGATCTCTTGTANN
PBGSF30 CTTGTA	Adapter primer with CTTGTA barcode	P-TACAAGAGATCGGAAGAGCGTCGTGTAGGGAAAGAGT-P
PBGSF29 CGTACG	Adapter primer with CGTACG barcode	TTCCCTACACGACGCTCTTCCGATCTCGTACGNN
PBGSF30 CGTACG	Adapter primer with CGTACG barcode	P-CGTACGAGATCGGAAGAGCGTCGTGTAGGGAAAGAGT-P
PBGSF23	GSF amplification primer 1	CAAGCAGAAGACGGCATACGAAGACCGGGGACTTATCATCCAACCTGT
PBGSF31	GSF amplification primer 2	AATGATACGGCGACCACCGAGATCTACACTCTTTCCCTACACGACGCTCTTCCGATCT

a For the primer sequences, the underlined sequence indicates the restriction enzyme site. For oligonucleotides for Tn-seq, the underlined sequence indicates the barcode sequence for demultiplexing. GSF, genome sequence footprinting (29).

Tn mutagenesis was performed by biparental mating using E. coli SM10-λpir containing pSAM_R1 or pSAM_R5 as a donor strain, as described previously (25). Briefly, 10 ml of a H. seropedicae culture was mixed with 5 ml of E. coli SM10-λpir (containing pSAM_R1 or pSAM_R5), both at an optical density at 600 nm (OD_600_) of 0.8. Bacterial cells were washed once with phosphate-buffered saline (PBS) (Sigma) and resuspended in 1.5 ml of PBS; 100 μl of this suspension was spotted on TY plates without antibiotics, left to dry, and incubated overnight at 30°C. Bacterial colonies were scraped from the plates and pooled in 10 ml of TY medium. One hundred microliters of this suspension was plated on TY agar with streptomycin, either kanamycin or tetracycline was added (depending on the resistance cassette in the Tn element used), and the plates were incubated overnight at 30°C. Bacterial colonies were scraped from the plates into 2 ml of TY medium per plate and pooled. The cell suspension was diluted in 50 ml of TY medium with the appropriate antibiotics, at an initial density of 1.5 × 10^7^ cells per ml (OD_600_ of 0.15), and was grown to an OD_600_ of 0.5, with shaking. Aliquots were mixed with glycerol to a final concentration of 15% and then were frozen at −80°C for future use.

As a quality control, the random insertion of transposons into the chromosome was analyzed using a single-primer PCR amplification approach to map the Tn insertion site, as described previously (27). Briefly, randomly selected colonies were isolated from the first Tn library, genomic DNA was extracted, and the Tn insertion site was amplified by PCR using the primer Km_RV2 (Table 2), followed by Sanger DNA sequencing of the amplicon. The sequence was aligned to the H. seropedicae SmR1 genome to identify the Tn insertion site.

### Characterization of Tn mutant libraries by Tn-seq.

Tn mutant libraries were characterized by Tn-seq, essentially as described previously (28, 29). Briefly, genomic DNA from Tn mutant libraries was isolated using the DNeasy blood and tissue kit (Qiagen) but, before the manufacturer's recommendations for Gram-negative bacteria were followed, cells were washed once with 1 M NaCl and once with PBS. Five micrograms of genomic DNA was digested with the restriction enzyme MmeI, and double-stranded Tn-seq DNA adapters with different barcodes were ligated to the restriction fragments. Tn insertion site flanking sequences were amplified by PCR using adapter- and mariner Tn-specific primers, using NEBNext Q5 High-Fidelity DNA polymerase (New England BioLabs). Cleanup of the PCR products was performed using MinElute PCR purification columns (Qiagen), DNA concentrations were measured with a Qubit fluorometer (Life Technologies), and DNA was sequenced using 50-bp single-end sequencing on a HiSeq 2500 Illumina sequencing platform (Genomics Core Facility at Cancer Research UK).

### Identification of genes essential for *in vitro* growth and survival.

Tn-seq Illumina sequence reads were demultiplexed using the FastX toolkit barcode splitter and were analyzed using the ESSENTIALS pipeline (30). The following analysis parameters were used in the ESSENTIALS analysis: sequence reads were aligned with a minimal match of 16 nucleotides, repeat regions were filtered, reads mapping to the 3′ end of the gene were removed, genomic position bias was corrected through Loess normalization, and read counts were normalized with the trimmed mean of *M* values (TMM) normalization method. In the implemented EdgeR statistical analysis part of ESSENTIALS, the dispersion was estimated with the Cox-Reid profile-adjusted likelihood method and the variance was modeled using common dispersion. To determine the number of unique Tn insertion mutants in each library, a read count cutoff value was derived from Kernel density plots in R, which allow delineation of “true” Tn insertions from “noise” sequencing reads. The distribution of Tn insertions was visualized by plotting the log_2_ read count for each chromosomal position, using an in-house Perl script. As a measure of gene essentiality, the log_2_ fold change between the observed and expected sequence reads was calculated for each gene, and a cutoff value was determined as described previously (30). Genes that had no informative TA insertion site flanking sequences (43 genes), i.e., no unique flanking sequences, were excluded from the analysis; for reference, these genes are listed in Table S1 in the supplemental material. Additional selection criteria were as follows: a *P* value adjusted with the Benjamini-Hochberg method of <0.05 and a probability that the gene was hit by a Tn of >0.95, as calculated using a derivative of Poisson's law, i.e., 1 − *e*^*N*^^×^^ln(1^^−^^*f*^^)^, with *N* being the number of unique Tn insertion mutants and *f* representing the number of unique TA flanking sequences in a gene divided by the number of unique TA flanking sequences in the genome. In addition, genes for which no sequence reads were detected and the probability of disruption was >0.95 were considered for further analysis. Analysis of functional class enrichment of candidate genes was performed using Fisher's exact test and was corrected for multiple testing using *Q* values (31). Genes required for *in vitro* growth and survival of H. seropedicae were visualized in DNAplotter (32).

### Determination of essential gene features.

A homology search of the Database of Essential Genes (DEG) (www.essentialgene.org) was performed using the BLASTP tool. Other analyses (Clusters of Orthologous Groups [COG] category assignment, metabolic pathway description, and prediction of transmembrane domains and signal peptides) were performed with tools of the Integrated Microbial Genomics platform (http://img.jgi.doe.gov) (33).

### Accession number(s).

Illumina Tn-seq sequencing data have been deposited in the European Nucleotide Archive (http://www.ebi.ac.uk/ena) and are available under accession number PRJEB15080.

## RESULTS AND DISCUSSION

### Characterization of H. seropedicae SmR1 Tn mutant libraries.

To identify genes critical for the growth of *H. seropedicae,* Tn mutant libraries were constructed under nutrient-rich conditions, i.e., in TY medium, using a biparental mating protocol. The *in vivo* Tn mutagenesis had an efficiency of ∼5 × 10^−6^ Tn mutants per H. seropedicae recipient cell. A total of six Tn mutant libraries were constructed, with sizes ranging between 24,000 and 140,000 CFU (Table 3).

**TABLE 3:** Tn mutant libraries constructed in H. seropedicae SmR1^*a*^

Library (antibiotic marker)^*b*^	Estimated Tn library size (CFU)	No. of sequence reads	No. (%) of aligned reads	No. (%) of insertion site flanking sequences hit in library	Average no. of reads/flanking sequence
A (Km)	24,000	14,008,866	10,465,200 (74.7)	26,590 (15.5)	394
B (Km)	55,000	3,046,565	2,491,660 (81.8)	19,038 (11.1)	131
C (Km)	90,000	25,122,546	22,284,725 (88.7)	52,327 (30.5)	426
D (Km)	140,000	20,353,571	18,537,039 (91.1)	50,639 (29.5)	366
E (Tc)	70,000	12,165,012	10,998,288 (90.4)	30,984 (18.0)	355
F (Tc)	50,000	7,775,781	6,969,727 (89.6)	28,492 (16.6)	245

a The criteria for identification of unique Tn insertion sites are listed in Materials and Methods.

b Km, kanamycin; Tc, tetracycline.

**FIG 1:**
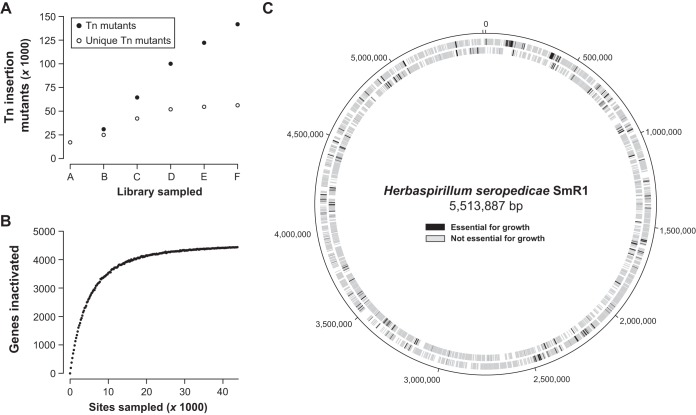
Characterization of H. seropedicae SmR1 Tn mutant libraries. (A) Cumulative numbers of Tn insertions in the different constructed mutant libraries and the numbers of unique Tn insertion mutants obtained. (B) Rarefaction analysis of intragenic Tn insertion positions, indicating near saturation of the number of genes that can be inactivated with a Tn. (C) Circular genome visualization, indicating the genes required for growth and survival in H. seropedicae SmR1.

Tn insertion site sequencing (Tn-seq) was performed using Illumina sequencing. Of the 88,320 potential TA dinucleotide mariner Tn insertion sites in the H. seropedicae SmR1 genome, 56,174 insertion sites (i.e., 63.6% of the total TA sites) were hit by a Tn insertion (Table 3). A cumulative analysis of amalgamating libraries revealed that the number of new unique Tn insertion mutants leveled off at ∼55,000 mutants (Fig. 1A). This suggests that, although we achieved Tn insertions in only ∼64% of the potential TA dinucleotide mariner Tn insertion sites, the maximum empirical number of mutants was obtained with this approach (without the use of much larger libraries). In addition, rarefaction analysis showed that we reached saturation in terms of the number of genes in the H. seropedicae genome that could be mutated (Fig. 1B). Tn insertions were distributed evenly throughout the chromosome, without any apparent evidence of hot spots, with an average of one Tn insertion every 95 bp (Fig. 1C).

**FIG 2:**
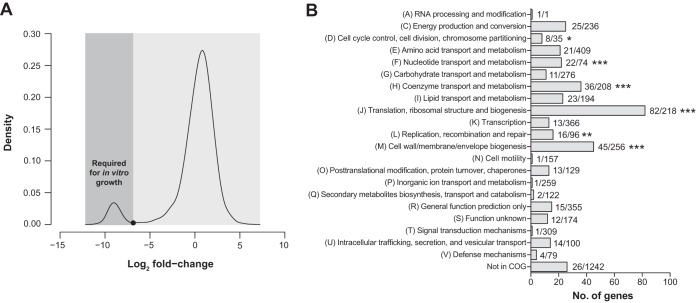
Identification and characterization of H. seropedicae SmR1 essential genes. (A) Density plot of log_2_ fold changes (measured reads/expected reads per gene). Black dot, gene essentiality cutoff value. (B) Functional class enrichment analysis of essential genes based on COG categories. Bars, number of essential genes assigned to each COG category, with the number of essential genes over the total number of genes in the COG category displayed to the right of each bar. COG category enrichment was analyzed using Fisher's exact test, with correction for multiple testing using *Q* values, as a measure of significance representing the false discovery rate (31). *, *Q* = 0.1; ∗∗, *Q* = 0.01; ∗∗∗, *Q* = 0.001.

It is widely assumed that genes with very few, or no, Tn insertions are essential for growth and survival or are underrepresented because their corresponding Tn insertion mutants have a growth defect (20) or they were not inactivated by a Tn element during Tn mutagenesis. To identify the genes required for growth under nutrient-rich conditions, a fold change was calculated between the actual number of sequence reads and the expected number of sequence reads (Fig. 2A); the latter takes into account the number of Tn mutants in the library, the length of the gene, and the number of possible Tn insertion positions (i.e., TA sites) for each gene (30). Of note, 43 genes lacked unique TA insertion site flanking sequences, and the essentiality of those genes could not be accurately addressed; the genes without unique TA flanking sequences are listed in Table S1 in the supplemental material. Analysis revealed that 136 genes had no reads at all and 296 genes showed log_2_ fold change (actual/expected sequence reads) values below −6.86. Next, to reduce the number of genes falsely identified as essential, we applied a 0.95 probability (calculated with a derivative of Poisson's law) cutoff value that the gene, if possible, was inactivated by a Tn insertion (based on 56,176 unique Tn mutants). Application of this cutoff value excluded 37 genes from the analysis, yielding a total of 395 genes that were found to be essential for *in vitro* growth and survival of H. seropedicae SmR1 in TY medium (see Table S2 in the supplemental material).

Essential genes were distributed relatively uniformly across the genome. However, eight regions larger than 100,000 bp were found to be dispensable for growth and survival. The two largest dispensable regions were located between Hsero_2418 and Hsero-4580 (*trnL*) (202,525 bp) and between Hsero_4426 (*glmS*) and Hsero_4580 (194,479 bp).

### In-depth analysis of the genes required for *in vitro* growth and survival.

Of the 395 genes identified as being required for growth and survival in TY medium, 22 corresponded to tRNA genes and 1 corresponded to a 23S rRNA gene (Hsero_4734 [*rrlC*]) (see Table S2 in the supplemental material). The other two 23S rRNA genes, i.e., *rrlA* (Hsero_0480) and *rrlB* (Hsero_3882), could not be evaluated for their essentiality as they had no unique TA insertion site flanking sequence (*rrlA*) or the probability of inactivation was only 0.632 (*rrlB*). Of the remaining 372 protein-coding genes required for *in vitro* growth, 346 were assigned a COG identifier. The COG categories significantly enriched among the genes identified as being essential in H. seropedicae are shown in Fig. 2B and included cell cycle control, cell division, and chromosome partitioning (category D); nucleotide transport and metabolism (category F); coenzyme transport and metabolism (category H); translation, ribosomal structure, and biogenesis (category J); replication, recombination, and repair (category L); and cell wall/membrane/envelope biogenesis (category M). The COG category of RNA processing and modification (category A) had only one representative, the product of the gene Hsero_1434, which is predicted to encode an oligoribonuclease. A total of 1,624 protein-coding genes containing transmembrane domains or signal peptides are present in the genome, and we identified 72 of those as being essential; 64 were assigned to one or more COG categories. As expected, the most represented COG category in this subset was cell wall/membrane/envelope biogenesis (category M).

### Essential metabolic pathways.

*In silico* analysis has revealed that H. seropedicae cannot utilize l-histidine, l-arginine, or l-lysine as carbon sources (8, 34). The l-histidine and l-lysine degradation pathways are incomplete, and no specific l-arginine transporter has been identified. In agreement with these findings, our Tn-seq data indicate that the genes involved in the biosynthesis pathways of these proteinogenic amino acids are essential. In addition, and to our knowledge not previously reported, both serine and glutamine synthesis seem to be essential for H. seropedicae growth in TY medium. In the case of glutamine, *glnA* (encoding glutamine synthetase) appears to be essential. Together with the glutamine oxoglutarate aminotransferase (GOGAT) enzyme, GlnA is the main route of assimilation of NH_4_^+^ in bacteria (35, 36) and, considering TY medium as a nitrogen-rich medium, we assume that GlnA activity should be low (36) and therefore nonessential under these conditions. GlnA activity and *glnA* expression were shown previously to be reduced but not absent when nitrogen levels were in excess of 20 mM NH_4_^+^ (37). No reduction in the expression of this gene or the activity of the enzyme was observed in the presence of glutamate (37). It is possible that nitrogen, from amino acids and peptides, may be more abundant in TY medium; hence, *glnA* is probably expressed and GlnA is active.

Another candidate essential gene related to nitrogen metabolism is *ntrX* (Hsero_0069), which encodes a two-component response regulator protein. Interestingly, a comparative genomics study reported that this gene is overrepresented in endophyte genomes, compared to the genomes of phytopathogens and rhizospheric bacteria (4).

We determined several genes encoding proteins in the pentose phosphate and glycolysis pathways to be essential. Three enzymes of the citric acid (tricarboxylic acid [TCA]) cycle, i.e., aconitate hydratase (*acnA* [Hsero_2979]), 2-oxoglutarate dehydrogenase components E1 (*sucA* [Hsero_2969]) and E3 (*lpdA* [Hsero_2967]), and two subunits of succinate dehydrogenase (*sdhB* [Hsero_2972] and *sdhC* [Hsero_2974]), were essential. Hsero_2971 (annotated as hypothetical) was also found to be essential; this gene has homology to *sdhE*, the product of which assists in the covalent attachment of flavin adenine dinucleotide (FAD) to SdhA (the product of the gene Hsero_2973) (38), which was not identified as essential.

Functional redundancy between genes precludes essentiality of central metabolic pathways; however, two homologous genes are not always redundant in their functions. In the case of the already mentioned *acnA* gene (Hsero_2979), which codes for the TCA cycle enzyme aconitate hydratase, H. seropedicae contains in its genome another gene annotated as *acnA* (Hsero_2283), with 41.89% identity. However, a mutant of that gene was identified in a Tn mutant library previously described for the closely related strain H. seropedicae Z67 (39); this suggests that *acnA* (Hsero_2283) does not participate in the TCA cycle. Homologs of *iscA* (Hsero_3845 and Hsero_3142), a gene involved in Fe-S cluster biogenesis, were identified as essential genes. The two genes belong to the same COG0316, pfam01521, and TIGR00049 families. Their essentiality indicates that they are not functionally redundant. This suggests the existence of different Fe-S biogenesis machineries for different proteins.

The *hfq* gene (Hsero_2948), encoding an RNA chaperone, is also essential for H. seropedicae SmR1 under the conditions studied. Several attempts to construct a defined deletion mutant of this gene were unsuccessful (Emmanuel de Souza, personal communication). The Hsero_4268 gene encodes a plasmid maintenance system antidote protein that we identified as being essential in our analysis. Transcriptome sequencing (RNA-seq) expression analysis showed that this gene and its toxin counterpart gene, Hsero_4269, were actively expressed in minimal medium (13), which indicates that there is an active toxin-antitoxin system in H. seropedicae SmR1.

### Critical reflection on identified candidate essential genes.

In this study, the Tn mutants were grown in pools. Consequently, Tn mutants with reduced fitness (i.e., slowly growing/dividing bacteria) would be present at lower abundance in the pools (reflected by lower read counts for Tn flanking sequences), and the corresponding genes could be tagged as essential in our analysis, i.e., the number of sequence reads per gene would fall below the essentiality cutoff value (21).

As part of our preliminary studies of the Tn libraries, we performed Sanger sequencing to identify the Tn insertion site in eight randomly selected mutants. Through this, we identified a mutant in which the Tn was inserted in the *dadX* gene (Hsero_2150). The enzyme encoded by this gene is predicted to catalyze the conversion of l-alanine to d-alanine, which then is incorporated into the peptidoglycan biosynthesis pathway by the d-alanine–d-alanine ligase protein (encoded by the gene *ddlB* [Hsero_0338]). Interestingly, according to our Tn-seq data, *dadX* appears to be essential in H. seropedicae (see Table S2 in the supplemental material). We hypothesized that d-alanine may be synthesized via an alternative pathway at a lower rate, allowing recovery of the mutant as a single colony but not after growth in a Tn mutant pool, during which there is competition between Tn mutants. We hypothesized that the alternative pathway could rely on Hsero_4778, which is predicted to encode d-alanine transaminase (EC:2.6.1.21), which catalyzes the interconversion of pyruvate and d-glutamate to d-alanine and 2-oxoglutarate.

### Comparative analysis of candidate essential genes and genes in other bacteria.

To identify orthologs of the 372 (including *dadX*) protein-encoding candidate essential genes in H. seropedicae, a BLASTP search was performed (*E* value cutoff of 1 × 10^−5^, with >30% sequence identity over >50% of the sequence length) against essential genes in 39 bacterial strains of 28 bacterial species present in the DEG (accessed in July 2016) (40). A total of 347 H. seropedicae SmR1 essential genes had at least one essential ortholog among the bacterial species present in the DEG. The 347 H. seropedicae SmR1 genes had 8,472 orthologs in the database (see Table S3 in the supplemental material). The high percentage of genes identified as essential in our study that were also described as being essential in other bacterial species reinforces the quality of our candidate essential gene set.

A total of 25 genes were uniquely essential in H. seropedicae SmR1, i.e., no essential orthologs were found in the DEG (see Table S4 in the supplemental material). Of the 20 essential proteins annotated as hypothetical, 14 are essential only in H. seropedicae. Three are proteins related to secretion systems; Hsero_0751 and Hsero_0943 are related to the type VI secretion system and Hsero_0804 is related to the type III secretion system of H. seropedicae. Type VI secretion systems are important for bacterial competition through contact-dependent killing of competitors (41). RNA-seq analysis of H. seropedicae grown in minimal medium or attached to maize roots showed that genes encoding the type III secretion system were not expressed in either case (13). This might suggest that Hsero_0804 is essential conditionally, i.e., when the bacteria are grown in nutrient-rich media.

Five of the genes uniquely essential in H. seropedicae code for transcriptional regulators, three of which belong to the transcription COG category. Hsero_1027 is homologous to the global regulator gene *pecS* from the phytopathogen Dickeya dadantii 33937, which is reported to repress the premature expression of virulence genes during the first stage of plant infection, when D. dadantii has to colonize the plant apoplast without provoking symptoms (42). A D. dadantii *pecS* mutant is hypervirulent (43). The expression of *pecS* is downregulated (fold change of −12.24; *P* = 9.39 × 10^−9^) in H. seropedicae attached to maize roots, implying that the genes repressed by PecS are expressed and may be important under those conditions (13). However, the products of those genes may be toxic when expressed under nutrient-rich conditions. The genes Hsero_1086, Hsero_2104, and Hsero_2356 code for transcriptional regulators with lambda-repressor-like, DNA-binding domains. Hsero_2356 is part of a locus (Hsero_2351 to Hsero_2371) that has a lower GC content (56% GC) than the rest of the SmR1 genome (63% GC). Interestingly, RNA-seq expression profiling of bacteria grown in minimal medium as well as bacteria attached to maize roots showed that genes of this locus (Hsero_2351 to Hsero_2356) were highly expressed, while the genes downstream of this genomic locus were not (13). We hypothesize that the essentiality of these three regulators could be due to repression of genes that might be lethal under the growth conditions used in our study. The gene Hsero_4425 is annotated as a member of the AsnC family of transcription-regulating proteins. It is divergently transcribed from the essential gene *glmS* (Hsero_4426). Homologs of *glmS* have been described as essential for 25 other bacterial species, and the arrangement of these two genes is conserved in many proteobacteria (data not shown). It is possible that the essentiality of Hsero_4425 in H. seropedicae SmR1 is related to the expression of *glmS*. Finally, the essential hypothetical genes Hsero_2418 and Hsero_3074 are both adjacent to genes coding for homologs of the RNA polymerase sigma E factor protein RpoE (Hsero_2419 and Hsero_3073). Both genes have predicted transmembrane helices; in the case of Hsero_2418, it belongs to the pFAM PF13490 family, i.e., a putative zinc finger found in several anti-sigma factor proteins. Homologs of these two genes are always linked to RNA polymerase sigma factors in other bacteria. We hypothesize that the essentiality of these genes in TY medium could be due to regulation of genes activated by the cognate sigma factors.

H. seropedicae candidate essential genes with described essential orthologs in only one or two of the strains in the DEG are indicated in Table S3 in the supplemental material. Interestingly, the gene Hsero_4295, which codes for an outer membrane porin, has essential orthologs only in the two betaproteobacteria Burkholderia thailandensis E264 and Burkholderia pseudomallei K96243 (44, 45), for which the essential gene sets have been described. Further, the gene Hsero_4295 was reported to be upregulated when H. seropedicae was attached to wheat roots but downregulated when H. seropedicae was attached to maize roots (12, 13), suggesting that this gene may be involved in host specificity. Six of the genes described in Table S3 were found to be essential only in H. seropedicae and in the soil inhabitant B. thailandensis. This subset of genes might indicate essential systems for Burkholderiales.

### Conclusions.

In this study, we have developed functional genomic techniques and resources for the model endophyte H. seropedicae that had not used previously in this species or in other bacterial endophytes. We have generated large comprehensive Tn libraries, and we have characterized the Tn insertion sites using next-generation sequencing (Tn-seq). These nearly saturated Tn libraries allowed us to perform robust essentiality analysis, and the results obtained are consistent with those reported for other bacteria. Our analysis of H. seropedicae Tn libraries from TY medium has enabled us to define the genes that are essential under those growth conditions. The results obtained enabled us to describe, at a functional level, the mechanisms of growth of H. seropedicae, including synthetic pathways, toxins, and regulatory mechanisms. Furthermore, these Tn libraries represent a valuable resource for the endophyte research community and will facilitate studies into the comprehensive assessment of the genetic mechanisms of the endophytic lifestyle of H. seropedicae, i.e., attachment to the root surface, internal colonization of the plant, and survival of the bacteria inside plants.

## Supplementary Material

Supplemental material
