# Supplementary material for: Essential Genes for In Vitro Growth of the Endophyte Herbaspirillum seropedicae SmR1 as Revealed by Transposon Insertion Site Sequencing
Source: Appl Environ Microbiol. 2016 Oct 27;82(22):6664–71. doi: 10.1128/AEM.02281-16 (PMC5086560; doi:10.1128/AEM.02281-16)
Supplement: Supplemental material [file supp_82_22_6664__index.html]

Supplemental material 

# Essential Genes for *In Vitro* Growth of the Endophyte Herbaspirillum seropedicae SmR1 as Revealed by Transposon Insertion Site Sequencing

## Supplemental material

- Supplemental file 1 -

  Distribution of Tn insertion sites in *H. seropedicae* strain SmR1 Tn mutant libraries (Fig. S1); supplemental table legends.

  PDF, 151K
- Supplemental file 2 -

  *H. seropedicae* SmR1 genes lacking unique TA insertion sites (Table S1).

  XLSX, 52K
- Supplemental file 3 -

  Overview Tn-seq analysis of genes essential for *in vitro* growth of *H. seropedicae* SmR1 (Table S2).

  XLSX, 315K
- Supplemental file 4 -

  Essential genes of *H. seropedicae* with homologs in the DEG database (Table S3).

  XLSX, 107K
- Supplemental file 5 -

  Essential genes of *H. seropedicae* SmR1 with no homologs in the DEG database (Table S4).

  XLSX, 10K
