# Supplementary material for: Essential Genes for In Vitro Growth of the Endophyte Herbaspirillum seropedicae SmR1 as Revealed by Transposon Insertion Site Sequencing
Source: Appl Environ Microbiol. 2016 Oct 27;82(22):6664–71. doi: 10.1128/AEM.02281-16 (PMC5086560; doi:10.1128/AEM.02281-16)
Supplement: Supplemental material [file AEM.02281-16_zam999117516so1.pdf]

SUPPLEMENTAL INFORMATION

FIGURE AND TABLE LEGENDS

Fig S1 Distribution of Tn insertion sites in *H. seropedicae* strain SmR1 Tn mutant libraries. Plotted are log_2 reads per Tn insertion above the read count cut-off (see Materials and Methods section).

Table S1 Genes lacking unique TA insertion sites

Table S2 Overview Tn-seq analysis of genes essential for *in vitro* growth of *H. seropedicae* SmR1.

Table S3 Essential genes of *H. seropedicae* SmR1 with homologs in the DEG.

Table S4 Essential genes of *H. seropedicae* SmR1 with no homologs in the DEG.

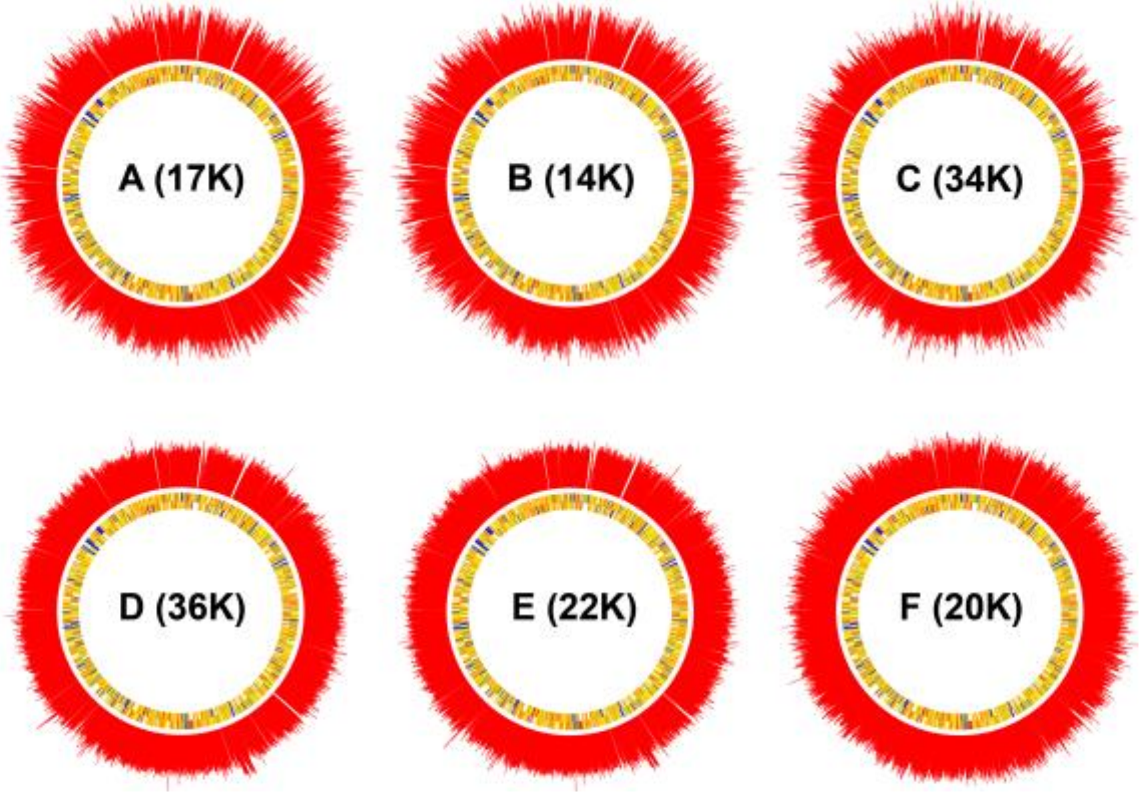

Fig S1
